# Gold

**Published:** 1861-01

**Authors:** W. Calvert


### DENTAL REGISTER OF THE WEST.

Vol. XV.] JANUARY, 1861. [No. 1.

### Original Essays and Communications.

### GOLD.

BY PROF. W. CALVERT, D. D. S.

Either the general or particular properties of this metal are too well known to the intelligent reader to be treated of here, my object moreover being rather to treat of the modus operandi of refining or purifying the same.

The process most commendable for dental Laboratory practice is the "humid" one. This consists in obtaining a solution of the metal through the direct agency of nitric and hydrochloric, or nitro-hydrochloric acid. The proportions of this Aqua Regia may vary from nearly equal parts of the two acids, to one part of nitric and four of hydrochloric.

When a metal is held in solution for the purpose of precipitation, the leading object should be to avoid a light, flocculent precipitate, and secure, if possible, a heavy and definitely crystalline deposit. Agreeably to the laws of crystallization, there are circumstances influencing, either favorably or unfavorably, the reduction of a metal when held by a solvent medium, such as heat and cold, the strength, or degree of the solution, both of the metal and the salt to be employed as a precipitant, as well as the rapid, or more gradual admixture of the two. These are things that should always be regarded, and, as well, the fact that equable results depend, in part, at least, upon the uniform strength of acids employed.

It is well known to those who are familiar with dissolving and precipitating gold, by the usual methods, that after the metal has been dissolved, the solution is diluted with water. Nothing, I am free to say, could be more prejudicial to the most desirable results than this. Again, it is not generally conceded to be a matter of any moment, whether the precipitant solution is of definite strength or not. Here, too, is a like error.

I might, furthermore, remark, that the indications I am about to give of the proportional admixture of acids, are applicable, especially to gold containing but comparatively a small amount of silver. Where silver and gold are combined, the silver being in large proportion, or in excess, another, and materially different method, should be adopted, as will be hereafter indicated.

Into a florence flask, then, put (by weight) of nitric acid, 38°B., twenty-five (25) parts, and of hydrochloric acid, 21° B., thirty (30) parts. This forms the desired *Aqua Regia*, or solvent.

The gold now to be dissolved should, if convenient, be laminated—the thinner the better—so that a large amount of surface may be exposed to the action of the acid, thereby hastening its solution. If coin is used, each piece should be curved in such a manner that, when in contact with each other, the broad, flat surfaces will not be in apposition, and thus retard the action of the acids. The metal being placed in the solvent medium, the whole should be exposed to the moderate heat of 100° F., of sand bath, until ebullition ceases, when, if the metal is not all dissolved, the solution may be poured off, and an additional quantity of fresh acid added, and the digesting by heat continued. It should be the aim, always, to have the acid take up all of the metal that it is capable of, or, in other words, to obtain a saturated solution. When the gold has all been dissolved, the metallic solution, being poured off, should all be placed in a suitable precipitating jar, and set aside to become cold.

As a precipitant, take the proto-sulphate of iron; and to prepare the solution, take any quantity of warm, or hot water, into which put this salt of iron until no more will dissolve—a saturated solution. This, now, should stand until quite cold.

When both solutions have become cold—and I might almost say, the colder the better—the proto-sulphate solution may be gradually added to the solution of gold, the result of which will be the falling of a heavy, brown precipitate—metallic gold. The solution of the salt of iron should be added as long as any precipitate continues to fall. When the precipitation is complete, and the whole has settled, the supernatant liquid may be decanted, or removed by means of a glass syphon. The gold should next be washed, by the addition of fresh, lukewarm water, the decanting, as before, and addition of water to be repeated, until it comes off free from acidity. After the last decanting of the water has taken place, the residue, together with all of the gold, should be removed to an evaporating dish, and, when no more water can be removed without loss of gold, the remaindor may be evaporated, leaving the pure gold in a highly crystalline powder, as may be readily seen under a medium magnifying power.

If desired, the metal may be packed into a clean crucible, placed in the fire, and subjected to a red heat for a short time, when it will have contracted very considerably, and, when cold, will be found susceptible of compression, and, subsequently, (due regard being had to annealing) may be wrought under the hammer into a massive form. This, however, being of but little practical utility, the proper course would be to fuse it in the crucible, the same having been previously well coated with borax, and, when thoroughly fused poured into the ingot mould.

I have already intimated that heat, cold, etc., influence the precipitation of a metal. Now, if either the metal or precipitant solution should be warm, it will be found that the deposit will not be so heavy as if it were cold; and, furthermore, will be darker in color. If the solutions be dilute, then the precipitate will be flocculent, and black. Should the iron be added very rapidly, the probability is that the deposit will be less heavy. And, lastly, if the acids employed are not of uniform strength, then, there will not be the same certainty of like results at different times.

I have already spoken of the refining of gold, and dwelt particularly upon the management of it when containing but a small amount of silver or alloy. My object now is to say something of refining the same metal, when there is a large proportion of silver present, or where the silver is in excess.

I may state here, that this method of treatment is that now practiced in the assay department of the United States mint.

The first step is to make an approximation, as to the relative proportion of silver in combination with the gold in hand for refining. This done, then add, by thoroughly fusing, an amount of silver (pure silver is preferable) sufficient to make the metals, when mixed, hold the relative proportions by weight of *one* part of gold to *two* parts of silver. When this combination has been effected in the crucible and cast in the ingot mould, it should be reduced or laminated into a thin sheet or ribbon—as thin as an ordinary mill will make it. This thin sheet should now be closely coiled or rolled up and placed in a suitable vessel to be subjected to the action of acid.

Unlike the former process, when aqua regia was employed for dissolving the gold, we here employ *pure dilute nitric acid.* The acid may now be poured over the coil of alloy, and a gentle heat applied, by which the action will be greatly promoted. This digesting should be continued until ebullition ceases, when, if there has been a sufficient quantity of acid, the silver, with copper, etc., will be entirely displaced, leaving the gold, in the original ribbon form, pure.

I would here take occasion to state that certainty, on the part of the manipulater, as to the cause of cessation in the action of the acid should be absolute—*not* that ebullition has ceased because of a saturation of the acid with silver, but rather from the entire displacements of the metal, which may be readily ascertained by the addition of a small quantity of fresh acid, and applying the heat again.

This process, as will be readily perceived, is based upon the principle of the excess metal being susceptible to the action of its proper solvent, and the fact that this *pure* acid is incapable of action upon the gold.

The acid holding the silver in solution should be poured off and retained, in order that the metal may be subsequently thrown down or reduced. The gold is now to be washed throughly with warm water, when it may be removed to a cupel, or to a piece of clean charcoal, and subjected to a cherry-red heat, after which it will be found in a pure state.

One advantage this method possesses over all others that I have any knowledge of, is that the gold remains united and entire, therefore lessening the liability of loss in the purifying process.

It is, I presume, the general rule of the laboratory to have suitable compartments for the reception of silver and gold *filings* which accrue in the ordinary routine of plate-work; yet, perhaps no arrangement will be so entirely secure as to prevent the admixture of the two metals in this form. In one case, the gold will be impoverished by the admixture of the silver; and, in the other, the silver will be uselessly enriched with the gold.

Here, then, comes into requisition the foregoing principle, in the employment of *pure* nitric acid to remove any silver that may have gained access to the gold, and in dissolving the silver filings, so as to make pure silver, thus leaving the filings of gold, that may have become mixed with the silver, untouched by the pure acid.

It will be observed that I have here laid some stress upon the use of *pure* nitric acid. It is necessary, therefore, that explanation be made as to the why and wherefore. When the acid in question is not pure, one of the common impurities is known to be hydrochloric acid, which combination favors the evolution of chlorine, the solvent principle of gold. Obviously, then, the *impure* nitric acid would result in a partial solution of the gold filings, and consequent loss of this metal.

